# Chronic lymphocytic leukemia with clinical debut as neurological involvement: a rare phenomenon and the need for better predictive markers

**DOI:** 10.1186/s12878-017-0073-0

**Published:** 2017-02-02

**Authors:** Cristhiam M. Rojas-Hernandez, Jacklyn Nemunaitis, Kristopher D. Marjon, Daniel Bustamante, Qian-Yun Zhang, Jennifer M. Gillette

**Affiliations:** 1 Division of Hematology and Oncology, University of New Mexico Health Sciences Center, Albuquerque, NM USA; 2 Department of Medicine, University of New Mexico Health Sciences Center, Albuquerque, NM USA; 3 Department of Pathology, University of New Mexico Health Sciences Center, Albuquerque, NM USA

**Keywords:** Leukemia, Central nervous system, CD49d, CD82

## Abstract

**Background:**

Chronic lymphocytic leukemia (CLL) is the most common leukemia in Western countries. The frequency of symptomatic central nervous system (CNS) involvement is unknown but thought to be a rare phenomenon. Currently there are no known risk factors for CNS involvement.

**Case presentation:**

We describe a clinically staged low-risk CLL case that presented with symptomatic CNS involvement and progressed rapidly to death. Evaluation of the surface adhesion molecules identified a markedly altered expression pattern of the integrin, CD49d, and the tetraspanin, CD82, in the index case when compared to similar low-risk CLL cases. We found that the early Rai clinical stage CLL patients showed linear correlation for the co-expression of CD82 and CD49d. In contrast, this unique index case with CNS involvement, which has the same Rai clinical stage, had a significantly lower expression of CD82 and higher expression of CD49d.

**Conclusions:**

These data suggest that the expression profile of CD49d and CD82 may represent potential biomarkers for patients with increased propensity of CNS involvement. Moreover, this study illustrates the critical need for a better mechanistic understanding of how specific adhesion proteins regulate the interactions between CLL cells and various tissue sites.

## Background

Chronic lymphocytic leukemia (CLL) is the most common hematologic malignancy in Western countries. The hallmark of CLL is the progressive accumulation of CD5+ B cells in the blood peripheral lymphoid organs, and bone marrow [1]. In rare cases CLL can involve the central nervous system (CNS). Recent systematic analysis data from reported cases in the literature and a large case series of over 4000 patients followed at a single institution have estimated a prevalence of symptomatic CNS involvement in 0.4% of patients with CLL, those findings are consistent with other previously reported antemortem studies [2–4]. In contrast, postmortem studies have demonstrated CNS involvement in up to 71% of patients with CLL [5]. Together, these data imply that CLL infiltration of the CNS is either underdiagnosed or frequently fails to manifest clinically. However, for CLL patients with symptomatic CNS involvement, the average time from diagnosis to death is 12 months [6].

In general, CLL follows an extremely variable clinical course with patient survival ranging from months to decades [4]. Several pathological markers exist for CLL diagnosis and prognosis, such as CD38 expression, immunoglobulin heavy chain variable region mutation status, and FISH panels for specific genetic alterations [6]. However, thus far, studies have failed to identify risk factors that effectively assist in early identification of CNS involvement [6]. There has been no strong correlation with age, sex, presenting symptoms, Rai stage, duration of disease, immunophenotype, or peripheral white blood cell (WBC) counts [5]. Initial studies reported that CNS infiltration occurs more frequently in late stage CLL [7, 8]. However, a more recent review of the literature indicates that one fourth of all the published cases of CNS involvement were during Rai stage 0 of CLL [6]. In fact recent data suggests that earlier CLL stages are more likely to present with neurological symptoms as the first signs of CLL, which is consistent with the case we present in this study. While 48.8% of patients with Rai stage 0-II disease presented with neurologic symptoms, only 10.7% of patients with stages III-IV disease presented with signs of neurologic involvement [6]. Therefore, the timely diagnosis of CNS involvement and the identification of risk factors for patients who develop CNS infiltration are crucial.

In combination, these data highlight the need for biomarkers that can predict CNS involvement. Soluble CD27 was a marker deemed useful at ruling out disease with a negative predictive value of 92%; however it was ultimately found to be highly non-specific with a positive predictive value of 54% [9]. As such there remains a critical need to identify specific markers that can be used to predict CNS involvement of CLL. We describe here a case that based on all clinical criteria, was staged as a low-risk CLL. Despite all of the low risk prognostic data, the patient presented with CNS involvement and progressed rapidly. Evaluation of the surface adhesion molecules on these CLL cells identified a unique differential expression pattern of the integrin, CD49d, and tetraspanin, CD82. Thus, analysis of the combined expression of CD49d and CD82 may serve as a potential indicator of CLL cells with propensity to involve the CNS.

## Case presentation

**Fig. 1:**
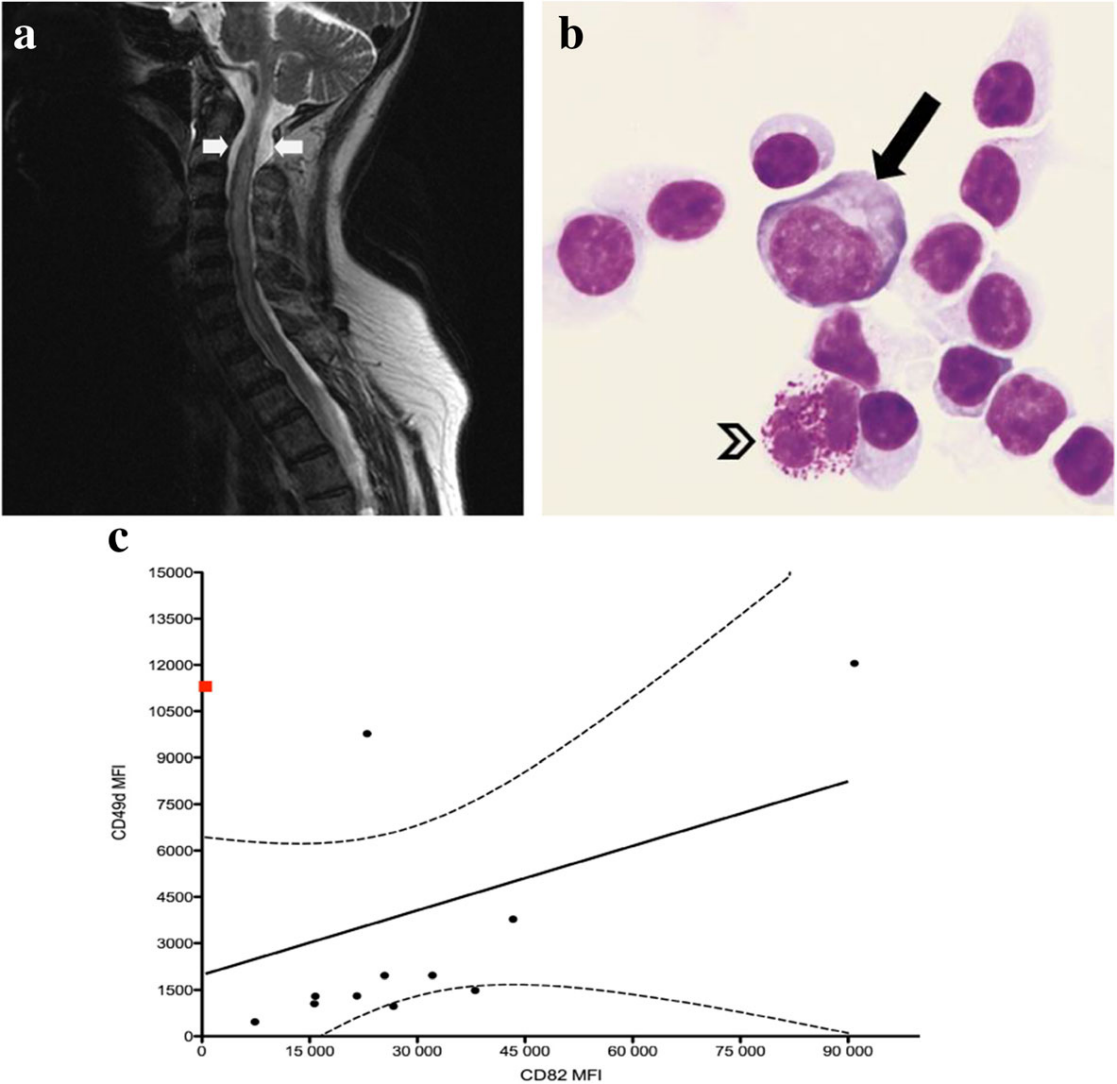
**a** T2 weighted sagittal cervical spine MRI evidencing segment enlargement and edema of the spinal cord extending from C2 to T1 levels (*arrows*). **b** Cytospin preparation of the cerebral spinal fluid (magnification × 1000, Wright stain) reveals a cluster of chronic lymphocytic leukemic cells with an admixed immunoblast (*solid arrow*) and a basophil (*open arrowhead*). **c** Correlation between CD82 and CD49d expression by mean florescence intensity detected (MFI) using flow cytometry in Rai clinical stage 0 CLL patients in the pilot study: linear trendline (*continuous line*) and 95% Confidence Interval (*interrupted line*). CNS case (*red square*) illustrated for comparison

A previously healthy 60 year old Caucasian female with no significant past medical history was admitted to the neurology service for progressive lower extremity weakness and urinary incontinence. She endorsed fever fatigue and a 20 pound weight loss over the preceding 3 to 4 months. Initially, the patient was presumptively treated for Parkinson’s disease with carbidopa/levodopa but had no improvement in symptoms. The patient denied any preceding trauma or any family history of neurological or hematological disease. The patient’s exam demonstrated 3/5 strength in the hip flexors and 4/5 strength in the remainder exam, hyperreflexia in the bilateral upper and lower extremities, and an otherwise normal neurological exam. No lymphadenopathy or splenomegaly was noted. A magnetic resonance imaging (MRI) of the brain and spinal cord was consistent with cord edema from C2 to T1 without evidence of a compressive lesion. Spinal cord MRI demonstrated no acute abnormalities (Fig. 1a). Serum antinuclear and anti-neutrophil cytoplasmic antibodies were not detected. Examination of the cerebral spinal fluid (CSF) demonstrated lymphocytosis (Fig. 1b). A comprehensive diagnostic investigation in the CSF and peripheral blood for bacterial, fungal, viral cultures and cryptococcal antigen did not reveal an infectious etiology. Oligoclonal banding in the CSF was not suggestive of multiple sclerosis. Additionally, flow cytometric analysis of the CSF (atraumatic and image-guided sample) and peripheral blood revealed a monoclonal B cell population that expressed CD19, dim CD20, dim surface light chain kappa, CD5, CD23, and lack of FMC7 expression. This immunophenotype was consistent with CLL. The complete peripheral blood cell count showed a lymphocyte count of 13400/uL, hemoglobin of 13 gr/dL, and platelet count of 362 000/uL. Lactate dehydrogenase level was 188 U/L. Staging imaging with CT of the chest, abdomen, and pelvis did not demonstrate splenomegaly or lymphadenopathy. As such, the case was identified as clinical Rai stage 0. The cytogenetics analysis by fluorescence in situ hybridization was negative for deletion of chromosomes 11, 13, 17 or aneuploidy for chromosome 12, the immunoglobulin variable region (*IGVH*) revealed a hypermutated status and the expression of CD38 was < 30% of the CLL cells by flow cytometric analysis. A multidisciplinary evaluation of this case suggested that this patient’s myriad of symptoms was the result of CLL with CNS involvement. The patient was started on rituximab 375 mg/m^2^ IV in combination with cyclophosphamide 250 mg/m^2^ IV on days 1–3 plus fludarabine 25 mg/m^2^ IV on days 1–3. After the first cycle of chemotherapy the patient exhibited improvement in the strength of the hip flexors with stable strength in the rest of her physical exam. No other new neurological abnormalities were noted at that time. She transferred to her hometown to establish care with a local oncologist. However, during the subsequent 8 weeks her performance status declined and the patient could not continue chemotherapy. She was eventually transitioned to comfort-based care in a hospice setting where her demise occurred.

## Discussion

We studied this case, nested in a pilot institutional project, evaluating the expression profile of specific adhesion molecules that regulate critical interactions between CLL cells and the microenvironment. Integrins, a family of cell adhesion molecules, play a significant role in cell motility, invasion and cell adhesion to the extracellular matrix and supportive stromal cells. In the case of CLL, the integrin CD49d or α4β1, has been linked to migration of CLL cells across the vascular endothelium and found to have clinical relevance for disease prognosis [10]. A recent study confirmed that patients with high CD49d expression had significantly increased CLL infiltration of the bone marrow [11]. Furthermore, a multicenter analysis demonstrated that patients with CLL expressing CD49d in ≥ 30% of neoplastic cells had a diminished 5 and 10-year overall survival and treatment free survival [11]. These findings were independent of CD38 and ZAP-70 expression. Finally, within a large-scale, worldwide, multicenter analysis, CD49d emerged as the strongest flow cytometry-based predictor of overall survival and treatment-free survival [12].

Interestingly, previous microscopy studies identified CD49d cellular localization within a protein complex enriched on the plasma membrane of CLL cells [10]. Recent work from our group established that CD49d protein organization and membrane density is regulated by the tetraspanin scaffold protein, CD82, which significantly impacts cell adhesion abilities [13]. Tetraspanins facilitate the formation of membrane protein complexes involved in multiple signaling pathways, tumor adhesion and dissemination [14]. CD82 is the protein product of the metastasis suppressor gene KAI1, which is located on chromosome 11p11.2. Several studies have confirmed its role as metastasis suppressor in many malignancies [15]. In patients with CLL, CD82 expression is upregulated when compared with peripheral blood mononuclear cells in normal controls. Moreover, a study evaluating gene expression in molecular subgroups of CLL found that CD82 was underexpressed in IGVH unmutated cases, suggesting a role for CD82 in clinically aggressive CLL [16]. However specific data on CD82 expression across different disease stages has not yet been described.

In our pilot study, CLL cells from peripheral blood were labeled in PAB buffer (PBS + 1% BSA + 0.02% sodium azide) for 30 min on ice with Alexa Fluor 647 CD82 (clone ASL-24; BioLegend) and Alexa Flour 488 integrin CD49d antibodies (clone 7.2R; R&D). Control tubes of cells were labeled with isotype controls: Alexa Flour 488 mouse IgG1 κ (clone 11711; R&D) and Alexa Flour 647 mouse IgG1 κ (clone MOPC-21; BioLegend). Cells were washed 3 times with PAB buffer and surface fluorescence was measured using an Accuri C6 flow cytometer. Control tube fluorescence values were subtracted from the experimental measurements to provide the mean fluorescence values indicated.

Subsequently, we performed correlation studies between the expression of CD49d and CD82 using a non-parametric measure of statistical dependence (Spearman’s rank correlation coefficient) to compare the mean fluorescence values of CD49d and CD82 in CLL patients with early Rai clinical stage, with a test significance level (*P* value) of 0.05. Our results on the early Rai clinical stage CLL patients (Table 1) showed linear correlation for the co-expression of CD82 and CD49d (Fig. 1c, Spearman *r* = 0.71, *P* < 0.05). However, in contrast, this unique index case with CNS involvement, which had the same Rai clinical stage, had a significantly lower expression of CD82 and higher expression of CD49d (Fig. 1c, red square). The enhanced expression of CD49d is consistent with poor patient prognosis in previous studies, where CLL cells with high CD49d expression showed enhanced trafficking and adhesion to the bone marrow along with increased cell survival [10–12]. Mechanistically, our previous data suggest that CD82 is a crucial regulator of CD49d membrane organization and molecular packing, which in turn modulates the adhesive potential of CD49d [13]. Therefore, the reduced expression of CD82 in this case may have disrupted the CD49d membrane packing or density, which would affect cell adhesion and migration abilities. We have shown that the membrane packing of CD49d is independent of its surface expression as detected by flow cytometric analysis. This shift in CD49d membrane organization may explain the reduced cell adhesion and enhanced migratory behavior of the CLL cells, which led to CNS invasion. Moreover, it is possible that the reduced expression of CD82 resulted in a disruption of metastasis suppression mechanisms, whereas the overexpression of CD49d promoted the extravascular migration of CLL cells to the CNS. Within our cohort of Rai stage 0 patients, the two additional patients with increased CD49d expression maintained indolent disease at the time of publication. Taken together, these data suggest that high CD49d expression might be coupled with reduced CD82 expression for enhanced predictive value.

**Table 1:** Demographic, clinical and prognostic characteristics of early clinical stage CLL patients

Characteristics	*n* = 14
Male	
*n* (%)	11 (79)
Age (years)	
Median (Min., Max.)	71 (60, 89)
Rai Clinical stage 0	
*n* (%)	14 (100)
Absence of B symptomatology	
*n* (%)	12 (86)
CNS disease	
*n* (%)	0
Hemoglobin (gr/dL)	
Median (Min., Max.)	14.6 (10.0, 17.1)
Platelet count (X10^3^ per microliter)	
Median (Min., Max.)	171 (128, 257)
Lymphocyte count (X10^3^ per microliter)	
Median (Min., Max.)	12.2 (4.0, 58.2)
Overexpression of CD38	
*n* (%)	3 (21)
Presence of IgVH hypermutation	
*n* (%)	2 (14)
Cytogenetic abnormalities: *n* (%)	
13q deletion	4 (29)
Trisomy 12	1 (7)
17p deletion	1 (7)
Follow-up duration from diagnosis (months)	
Median (Min., Max.)	74.8 (32.5, 188.6)
Progression to treatment	
*n* (%)	3 (21)
Time to progression from diagnosis (months)	
Median (Min., Max.)	59.1 (11.4, 66.5)

Despite the favorable risk features in our patient measured by conventional clinical (Rai staging) immunophenotypic and cytogenetic criteria; she presented with CNS involvement and neurologic symptoms. When comparing the adhesion molecular profile of this patient to the cohort of Rai stage 0 patients in our study we identified an inverse relationship between the expression of CD49d and CD82. With our current findings, we cannot confirm whether the change in expression of CD49d and CD82 in our case was a cause or an effect of CNS involvement. However, we hypothesize that the reduction of CD82 expression and the high level of CD49d may contribute to the CNS involvement and the poor patient outcome observed in this CLL case.

## Conclusions

This case highlights the need for better predictive markers in CLL, especially in atypical cases such as those with CNS involvement. Our data suggest that the expression profile of CD49d and CD82 may represent potential biomarkers in predicting CNS involvement of CLL. Furthermore, this study illustrates the critical need for a better mechanistic understanding of how specific adhesion proteins regulate the interactions between CLL cells and various tissue sites. We anticipate that future large-scale studies will help to elucidate dynamic changes in CD49d and CD82 expression across different tissues, disease stages and over the course of disease progression.

## Abbreviations

BSA: Bovine serum albumin
CLL: Chronic lymphocytic leukemia
CNS: Central nervous system
CSF: Cerebral spinal fluid
MRI: Magnetic resonance imaging
PBS: Phosphate-buffered saline
WBC: White blood cells
